# Cardiac dysregulation following intrahippocampal kainate-induced status epilepticus

**DOI:** 10.1038/s41598-020-60324-8

**Published:** 2020-03-04

**Authors:** Amber T. Levine, Heather A. Born, Andrew P. Landstrom, Samuel Larson, Wai Ling Lee, An T. Dao, Xander H. Wehrens, Yi-Chen Lai, Anne E. Anderson

**Affiliations:** 1 Department of Neuroscience, Baylor College of Medicine, Houston, TX USA; 2 Department of Pediatrics, Baylor College of Medicine, Houston, TX USA; 3 Department of Molecular Physiology and Biophysics, Baylor College of Medicine, Houston, TX USA; 4 Department of Neurology, Baylor College of Medicine, Houston, TX USA; 5 The Jan and Dan Duncan Neurological Research Institute, Texas Children’s Hospital, Houston, Texas USA; 6 The Gordon and Mary Cain Pediatric Neurology Research Foundation Laboratories, Texas Children’s Hospital, Houston, Texas USA; 7 Department of Pediatrics, Division of Cardiology, Duke University School of Medicine, Durham, NC USA

**Keywords:** Epilepsy, Cardiovascular biology, Neurophysiology

## Abstract

Status epilepticus (SE) is a prevalent disorder associated with significant morbidity, including the development of epilepsy and mortality. Cardiac arrhythmias (i.e. inappropriate sinus tachycardia and bradycardia, asystole, and atrioventricular blocks) are observed in patients following SE. We characterized ictal (during a seizure) and interictal (between seizure) cardiac arrhythmogenesis following SE using continuous electrocardiography and video electroencephalography (vEEG) recordings throughout a 14-day monitoring period in an intrahippocampal chemoconvulsant mouse model that develops epilepsy. We quantified heart rhythm abnormalities and examined whether the frequency of cardiac events correlated with epileptiform activity, circadian (light/dark) cycle, the presence of seizures, and survival during this period of early epileptogenesis (the development of epilepsy) following SE. Shortly following SE, mice developed an increased interictal heart rate and heart rhythm abnormalities (i.e. sinus pause and sinus arrhythmias) when compared to control mice. Heart rhythm abnormalities were more frequent during the light cycle and were not correlated with increased epileptiform activity or seizure frequency. Finally, SE animals had early mortality, and a death event captured during vEEG recording demonstrated severe bradycardia prior to death. These cardiac changes occurred within 14 days after SE and may represent an early risk factor for sudden death following SE.

## Introduction

Status epilepticus (SE) is a prolonged seizure event and affects roughly 10–41 per 100,000 individuals per year, with half of these events occurring when there is no prior history of epilepsy^1^. SE is associated with many comorbidities, including permanent neuronal damage, cognitive dysfunction, and increased risk for developing epilepsy in individuals with no prior epilepsy history who then experienced SE^1,2^.

During the period following SE, a variety of ictal (during a seizure event) and interictal (period between seizures) cardiovascular alterations have been described in humans and animal models. Studies in genetic and induced epilepsy animal models have shown altered cardiac repolarization, sinus tachycardia (elevated heart rate), and various cardiac arrhythmias during the interictal state^3,4^. During ictal periods, reports have shown tachycardia, bradycardia, myocardial ion channel dysregulation, and heart rate (HR) instability^5–8^. In addition to cardiac electrophysiological dysfunction, SE and epilepsy animal models have shown a variety of channelopathies, all of which have the potential to disrupt the normally precise timing mechanisms in the myocardium leading to potentially fatal arrhythmias^4,9,10^. These findings suggest unstable cardiac repolarization and increased arrhythmogenic potential following SE^9,11–13^.

Prior studies have not included a continuous assessment of cardiac electrophysiological activity in the context of altered electroencephalogram (EEG) activity that occurs following SE and early in the development of epilepsy (epileptogenesis). The goal of the current studies was to thoroughly define the development and progression of cardiac dysfunction during the early period (14 days [d]) following SE using continuous, synchronized video EEG (vEEG) and electrocardiography (ECG) monitoring^14,15^. We induced SE by delivering kainate through an intrahippocampal cannula to avoid systemic exposure to the chemoconvulsant and allow for direct hippocampal administration in freely moving, unanesthetized, and awake mice. Using this model, we recorded ECG and vEEG with continuous (24 hours [h]) monitoring at baseline, during SE induction, and for 14 days following SE. We quantified seizures, cardiac measures of variation in HR, any arrhythmic activity, and interval differences in PR (a measure of atrioventricular conduction speed), QRS (time for ventricles to depolarize), and QTc (a measure of ventricular repolarization that is corrected for the HR at the time of measurement).

## Results

We verified electrode and intrahippocampal cannula placement, as well as fluid dispersion from the intrahippocampal cannula (Supplemental Fig. 1a). Mice were placed in the recording chamber and recorded using synchronized vEEG and ECG (Supplemental Fig. 1b). To establish a baseline for the model, we recorded animals for 24 h prior to infusion of saline (Veh) or kainate (KA; induction). At baseline, animals did not show signs of epileptiform activity or cardiac arrhythmias (Supplementary Fig. 1c). With the infusion of kainate or saline, we evaluated vEEG for any behavioral seizures (scored using a modified Racine scale – see methods) and electrographic epileptiform activity (defined as the presence of spikes and seizure activity). ECG signals were monitored for the presence of cardiac rhythm disturbances. As expected, the saline-treated control animals had no changes in EEG activity when compared to baseline. However, all the animals that received kainate developed SE (Supplementary Fig. 1d).

To determine if electrical conduction through the heart was modified at 1 h and 24 h following SE, we analyzed the PR, QRS, and QTc intervals for 10 s of the ECG recordings at baseline and following SE^14,15^. We found no difference between Veh and SE animals in PR (Supplemental Fig. 1e; 1 h Veh: 35.41 ± 0.46 ms, SE: 37.44 ± 0.74 ms, 24 h Veh: 35.09 ± 0.90 ms, SE: 35.35 ± 0.83 ms, *P* = 0.40), QRS (Supplemental Fig. 1f; 1 h Veh: 9.53 ± 0.52 ms, SE: 9.80 ± 0.30 ms, 24 h Veh: 9.99 ± 0.43 ms, SE: 10.03 ± 0.27 ms, *P* = 0.82), and QTc (Supplemental Fig. 1g; 1 h Veh: 67.92 ± 5.14 ms, SE: 69.34 ± 3.63 ms, 24 h Veh: 58.61 ± 3.05 ms, SE: 67.20 ± 4.25 ms, *P* = 0.38) duration at 1 h and 24 h post-Veh or KA infusion.

### Sinus tachycardia and sinus nodal arrhythmias occur early following SE

**Figure 1:**
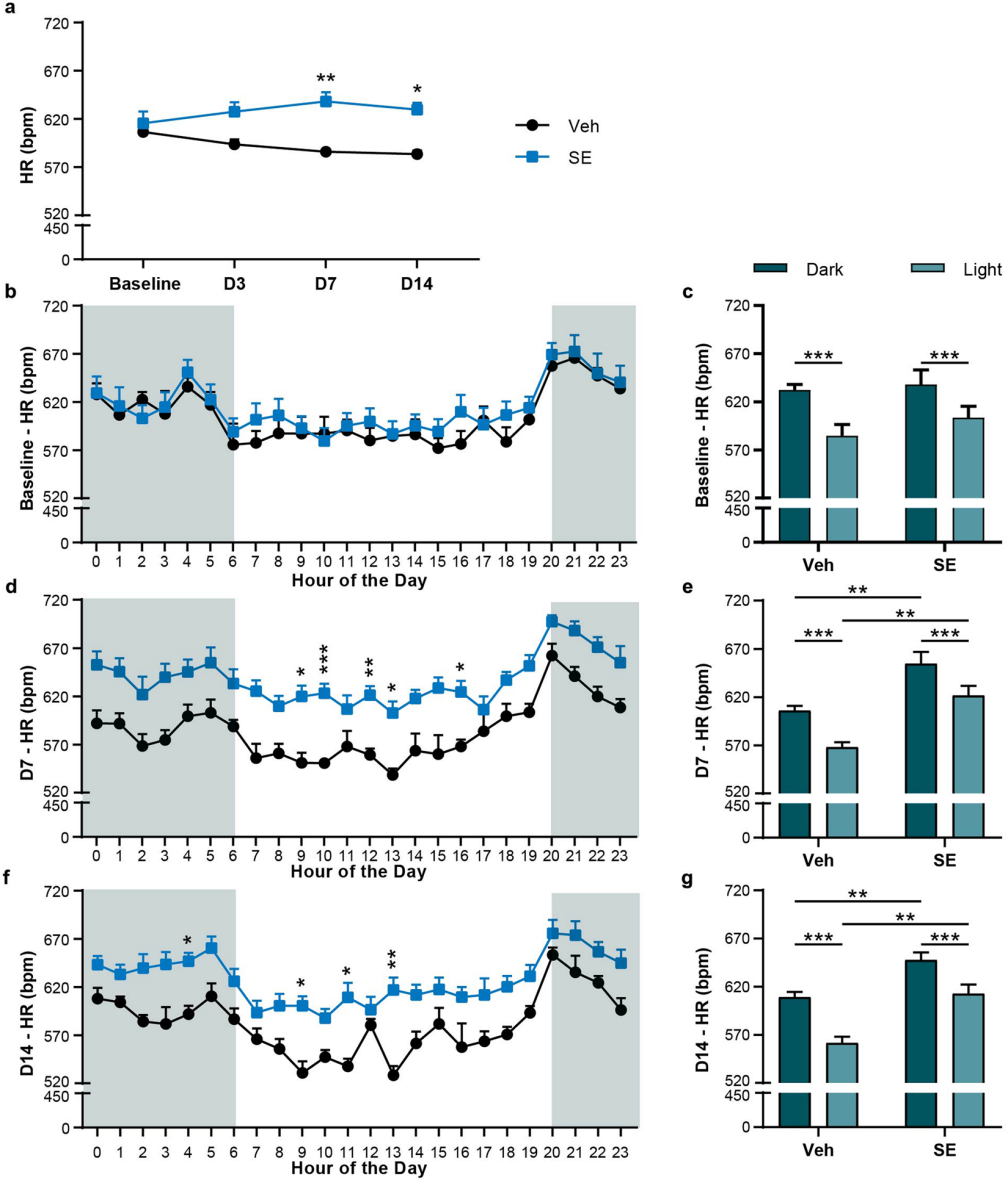
Interictal sinus tachycardia following SE. (**a**) Average HR, in beats per minute (bpm), over a 24 h period for Veh and SE animals at baseline, D3, D7, and D14 post-SE. On D7 and D14, SE animals had a significantly higher HR compared to Veh animals (D7, *P* = 0.003; D14, *P* = 0.011). (**b**) Veh and SE animals demonstrated no difference in average HR over the full 24 h of baseline recording (*P* = 0.96, two-way ANOVA). (**c**) Veh and SE animals showed no difference in baseline HR when the dark and light cycles were compared across groups (*P* = 0.48; two-way ANOVA). Both Veh and SE did show a difference in their respective light/dark cycle (Veh light vs dark, *P* = 0.0003; SE light vs dark, *P* < 0.0001). (**d**) At D7, SE animals showed an elevated HR over the course of the day with post-hoc differences at hours 9 (*P* = 0.021), 10 (*P* = 0.0008), 12 (*P* = 0.002), 13 (*P* = 0.010), and 16 (*P* = 0.023). (**e**) SE animals also had a higher HR during both the light (*P* = 0.003) and dark cycles (*P* = 0.005) compared to Veh animals. Veh and SE groups maintained a difference in their respective light/dark cycle comparisons (Veh light vs dark, *P* = 0.0005; SE light vs dark, *P* < 0.0001). (**f**) At D14, SE animals continued to show an elevated HR with post-hoc differences at hours 4 (*P* = 0.020), 9 (*P* = 0.035), 11 (*P* = 0.025), and 13 (*P* = 0.0024). (**g**) SE animals demonstrated a higher HR during the light (*P* = 0.0023) and dark cycles (*P* = 0.0062) compared to Veh animals, while both groups maintained their respective light/dark cycle differences (Veh light vs dark, *P* = 0.003; SE light vs dark, *P* = 0.003). Lightly shaded areas in b, d, and f indicate lights off. Unless noted, a two-way ANOVA with Sidak’s multiple comparisons test was used. All data points represent mean ± SEM (Veh: n = 5; SE: n = 10; *P < 0.05, **P < 0.01, ***P < 0.001).

To determine whether HR alterations developed and progressed during the early period following SE, we assessed interictal HR (measured in beats per minute [bpm]) and cardiac rhythms at baseline (prior to KA), 3, 7, and 14 days (D3, D7, and D14, respectively) post-SE. We found no difference in either the average 24 h (Fig. 1a) or hourly (Fig. 1b) HR between control and SE animals at baseline prior to KA (Veh: 606.51 ± 8.72 bpm, SE: 615.61 ± 12.30 bpm, *P* = 0.96) or at D3 (Fig. 1a; Veh: 593.74 ± 5.09 bpm, SE: 627.58 ± 9.65, *P* = 0.099). At baseline, both Veh and SE animals had a higher HR during the time period when the lights were off and the animals were more active (dark cycle) compared to when the lights were on (light cycle) and the animals were presumed to spend more time sleeping (Fig. 1c; Veh: dark [632.20 ± 5.85 bpm] vs light [584.74 ± 11.69 bpm], *P* = 0.0003; SE: dark [636.87 ± 13.51 bpm] vs light [597.36 ± 12.33 bpm], *P* < 0.0001). Veh and SE animals did not differ in the average HR during the dark and light time intervals (*P* = 0.48). When compared to Veh animals at D7 and D14, SE animals had a significantly higher average 24 h HR (Fig. 1a; D7, Veh: 586.04 ± 3.83 bpm, SE: 638.29 ± 9.53 bpm*, P* = 0.0033; D14, Veh: 583.43 ± 4.05 bpm, SE: 629.63 ± 7.30 bpm, *P* = 0.011). At D7, SE animals experienced sinus tachycardia at hours 9 (Veh: 550.97 ± 10.57 bpm, SE: 619.87 ± 11.36 bpm, *P* = 0.021), 10 (Veh: 550.71 ± 2.52, SE: 623.18 ± 9.94 bpm, *P* = 0.0008), 12 (Veh: 559.27 ± 6.58 bpm, SE: 621.44 ± 9.10 bpm, *P* = 0.002), 13 (Fig. 1d; Veh: 538.45 ± 6.70 bpm, SE: 602.77 ± 11.91 bpm, *P* = 0.01), and 16 (Veh: 567.96 ± 7.27, SE: 624.76 ± 11.28, *P* = 0.023). Both Veh and SE animals had a higher HR during the dark cycle when compared to the light cycle (Fig. 1e; Veh: dark [606.16 ± 4.67 bpm] vs light [568.00 ± 5.20 bpm], *P* = 0.0005; SE: dark [657.30 ± 11.42 bpm] vs light [622.10 ± 8.98 bpm], *P* = 0.0003) consistent with expected increased activity. SE animals experienced a higher HR during both dark and light time periods on D7 (dark: *P* = 0.005; light: *P* = 0.003). Additionally, at D14, SE animals experienced sinus tachycardia specifically at hours 4 (Veh: 591.92 ± 8.57 bpm, SE: 646.79 ± 8.64 bpm, *P* = 0.020), 9 (Veh: 530.82 ± 12.14 bpm, SE: 600.56 ± 9.89 bpm, *P* = 0.035), 11 (Veh: 537.60 ± 8.06 bpm, SE: 609.35 ± 14.92 bpm, *P* = 0.025), and 13 (Fig. 1f; Veh: 528.40 ± 9.65 bpm, SE: 616.99 ± 12.76 bpm, *P* = 0.0024). Again at D14, Veh and SE animals had a higher HR during the dark compared to the light time period [Fig. 1g; Veh: dark [608.97 ± 5.43 bpm] vs light [561.50 ± 6.25 bpm], *P* = 0.0023; SE: dark [651.84 ± 8.33 bpm] vs light [609.52 ± 9.01 bpm], *P* = 0.0003). Furthermore, compared to Veh animals, SE animals experienced a higher HR during both dark and light time periods on D14 (dark: *P* = 0.006; light: *P* = 0.003).

In addition to HR measures, we assessed whether cardiac electrical conduction was modified interictally. We found no difference in PR (Supplemental Fig. 2a, *P* = 0.77), QRS (Supplemental Fig. 2b, *P* = 0.56), or QTc (Supplemental Fig. 2c, *P* = 0.92) intervals when SE animals were compared to Veh at baseline, D3, D7, and D14 following SE. Additionally, we measured heart rate variability (HRV) at baseline (Supplemental Fig. 3a–c), and interictally on D3 (Supplemental Fig. 3d–f), D7 (Supplemental Fig. 3g,h,i) and D14 (Supplemental Fig. 3j,k,i) during both the light and dark cycles. No significant difference was found.

**Figure 2:**
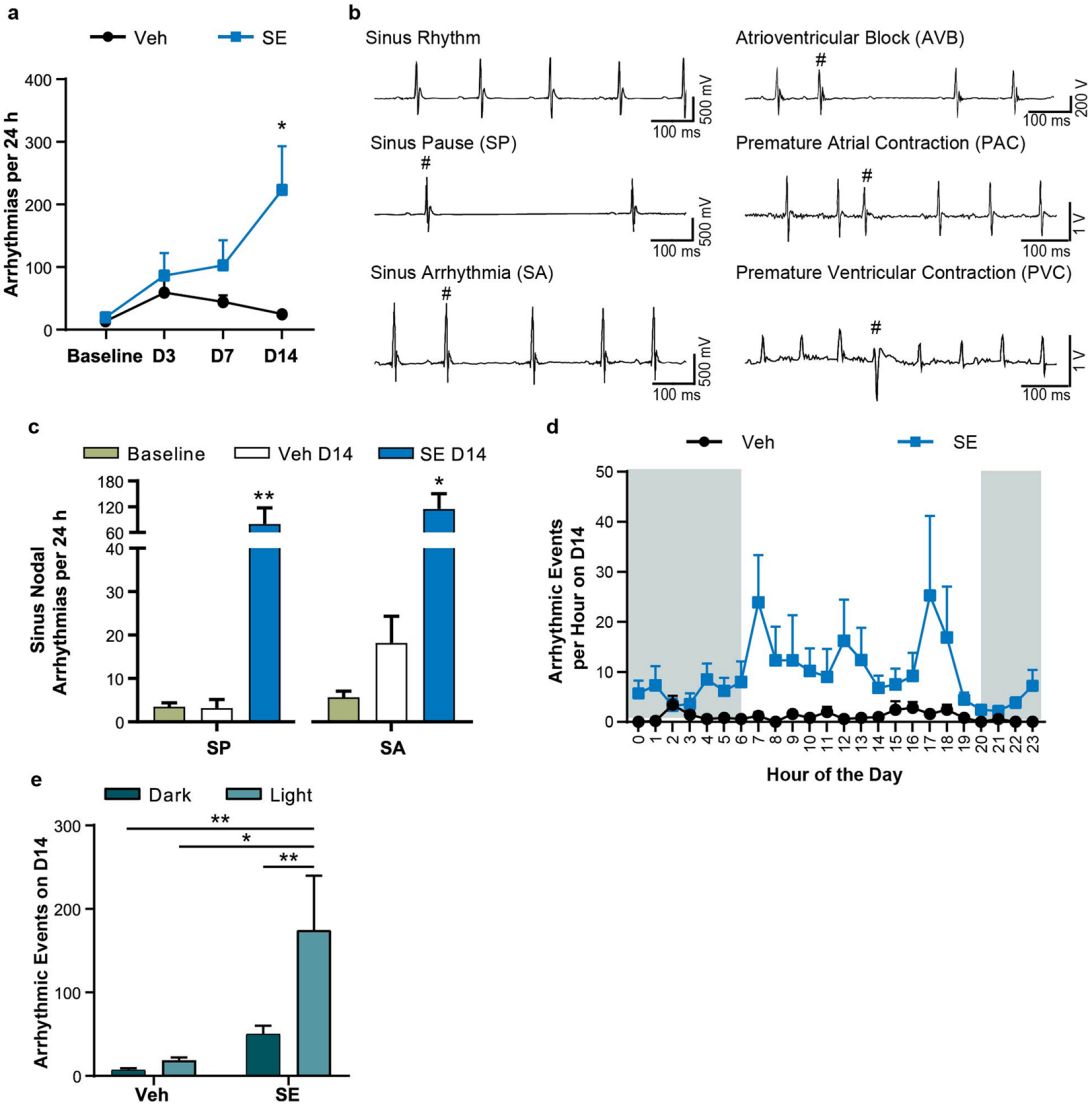
Cardiac rhythm abnormalities present interictally following SE. (**a**) SE animals showed an increased number of total arrhythmic events at D14 (*P* = 0.0127) but not during baseline, D3, or D7. (**b**) Examples of sinus rhythm, AVB, SP, PAC, SA, and PVC arrhythmias are shown and indicated by #. (**c**) By D14, SE animals developed an increased number of SP and SA events demonstrating an increased frequency of arrhythmic events compared to baseline (*P* = 0.0015) and Veh animals (*P* = 0.023). (**d**) The average number of combined arrhythmic events for each hour on D14 for SE and Veh animals. (**e**) Arrhythmic events occurred more frequently during the light cycle in SE animals compared to the dark cycle at D14 (*P* = 0.0014) or the Veh dark (*P* = 0.0094) and light cycles (*P* = 0.019). Comparisons were made using a two-way ANOVA with Sidak’s multiple comparisons test. All data points represent mean ± SEM (Veh: n = 5; SE: n = 10; *P < 0.05, **P < 0.01).

In parallel with the above analyses, we quantified heart rhythm abnormalities in ECG morphology at baseline, D3, D7, and D14. SE animals did not exhibit significant alterations in heart rhythm at D3 or D7 post-SE (Fig. 2a; D3, Veh: 59.4 ± 26.92, SE: 86.50 ± 35.74, *P* = 0.99; D7, Veh: 44.40 ± 10.44, SE: 103.00 ± 39.98, *P* = 0.838). On D14, the SE animals displayed a significantly increased number of heart rhythm abnormalities when compared to Veh animals (Fig. 2a; D14, Veh: 24.80 ± 4.67, SE: 223.50 ± 69.57, *P* = 0.013). We further categorized cardiac arrhythmias seen after SE as follows: atrioventricular block (AVB), premature atrial contraction (PAC), premature ventricular contraction (PVC), sinus pause (SP), and sinus arrhythmia (SA; Fig. 2b). No complex arrhythmias (i.e. atrial or ventricular flutter/fibrillation) were found in this model. SE animals had a significantly increased number of SP and SA events at 14 days when compared to Veh animals (Fig. 2c; SP, Baseline: 3.47 ± 0.94, Veh D14: 3.20 ± 1.98, SE D14: 79.50 ± 38.16, *P* = 0.022; SA, Baseline: 5.67 ± 1.43, Veh D14: 18.20 ± 6.15, SE D14: 114.90 ± 35.67, *P* = 0.031).

We evaluated whether the number of arrhythmic events was different depending on the time of day (Fig. 2d). SE animals experienced significantly more events during the light cycle compared to the dark cycle at D14 (Fig. 2e; dark: 50.10 ± 9.91 vs light: 174.40 ± 65.36, *P* = 0.0014). Furthermore, SE animals demonstrated a significant increase in arrhythmic events at D14 during the light cycle compared to Veh animals during either dark or light cycles (Fig. 2e; Veh dark: 7.00 ± 2.30, vs SE light: *P* = 0.0094, Veh light: 18.60 ± 3.70, vs SE light: *P* = 0.019).

### Epileptiform activity does not correlate with the number of arrhythmic events

To determine if epileptiform activity correlates with the number of arrhythmic events early following SE, we compared the presence of epileptiform activity with the frequency of heart rhythm abnormalities. Animals were monitored for spontaneous EEG changes and behavioral seizure activity to determine if any animals developed epilepsy within the 14-day monitoring period following SE. Neither Veh nor SE animals experienced behavioral or electrographic spontaneous seizures before or on D3. By D7 and D14 post-induction, 41% (5/12) and 50% (6/12) of SE animals developed spontaneous seizures, whereas Veh animals remained seizure free. Spontaneous seizures were characterized by at least 10 s of repetitive spiking or spike-and-slow wave activity and accompanied by motor behavior (hind/fore-limb clonus, and tonic posturing) indicative of a generalized tonic-clonic (GTC) seizure classification.

**Figure 3:**
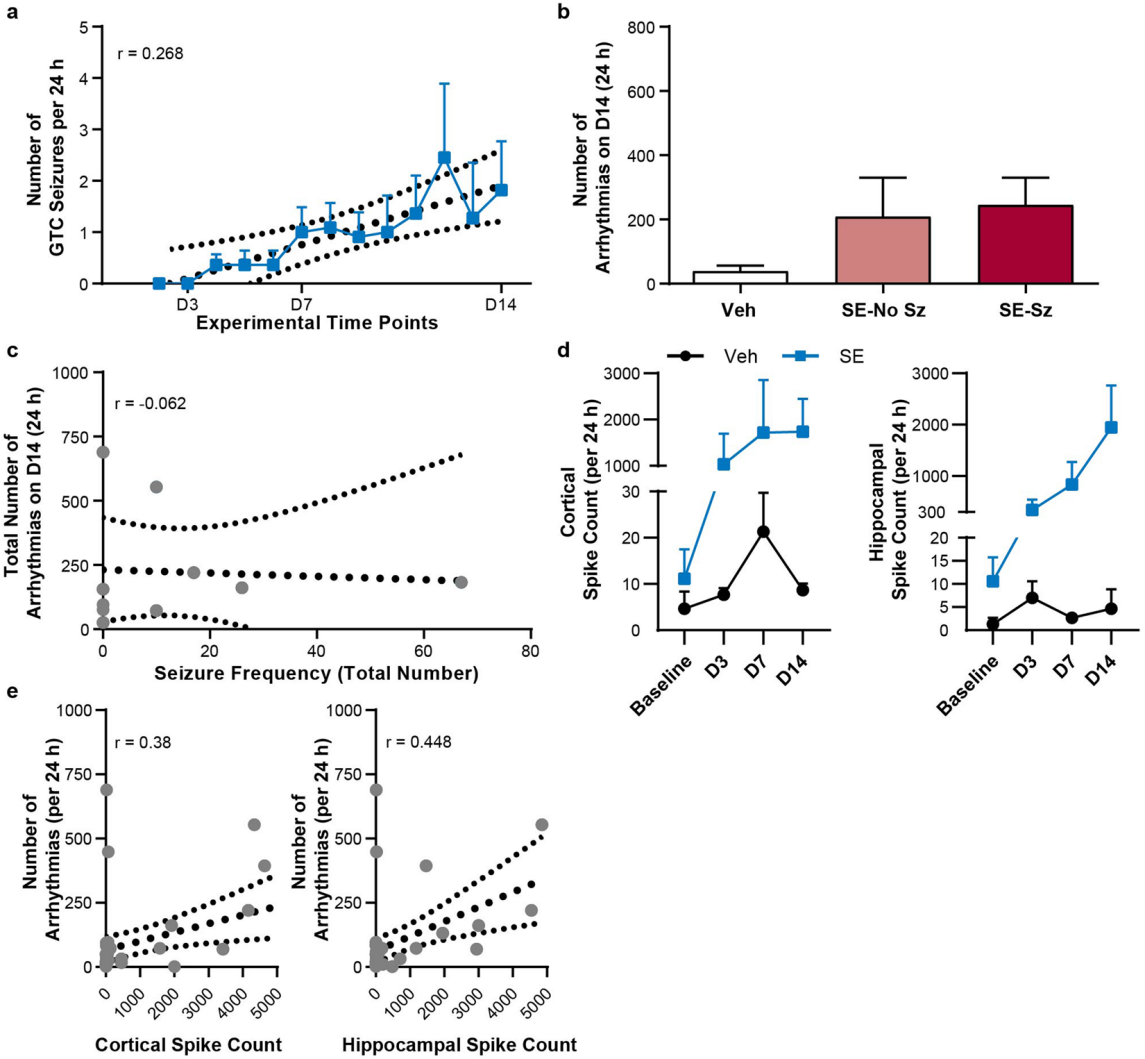
The frequency of epileptiform activity does not correlate with the number of cardiac arrhythmic events. (**a**) The average number of spontaneous seizures in SE animals over the 14-day monitoring period (r = 0.27; n = 10). (**b**) There was no difference in the number of arrhythmic events for Veh, SE-No Sz, and SE-Sz groups (*P* = 0.24; ordinary one-way ANOVA; n = 5 mice/group). (**c**) The number of arrhythmias at D14 did not correlate with seizure frequency in SE animals (r = −0.062; n = 10). (**d**) The average number of cortical and hippocampal spikes per 24 h in Veh (n = 3) and SE (n = 7) animals for baseline, D3, D7, and D14 showed no statistical difference (cortical, *P* = 0.57; hippocampal, *P* = 0.14; two-way ANOVA). (**e**) Neither cortical (r = 0.38; n = 40) nor hippocampal (r = 0.448; n = 40) spike frequency correlated with the total number of arrhythmias for Veh or SE animals. Linear regression lines and 95% confidence intervals are shown for all correlative analyses with dotted lines (**a**,**c**,**e**). a, b, and d represent mean ± SEM. Each symbol represents an individual animal in (**c**,**e**).

To determine whether the cardiac abnormalities correlated with ongoing epileptiform activity, the number of GTC seizures and arrhythmic events were compared. The frequency of daily GTC seizures was quantified for each animal (Supplemental Fig. 4). Similarly, the number of cortical and hippocampal interictal spikes were quantified at baseline, D3, D7, and D14 for the SE animals. We found increased seizure frequency for the SE animals that developed seizures (Sz) during the 14-day monitoring period (Fig. 3a). SE animals were then grouped according to the presence (SE-Sz) or absence (SE-No Sz) of seizure activity during the 14-day monitoring period to determine whether seizure burden following SE contributed to the number of arrhythmic events. No significant difference was observed in the number of arrhythmic events amongst SE-No Sz and SE-Sz groups at D14, suggesting that at this time point, previously undergoing SE rather than the current seizure burden was the critical risk factor for arrhythmic cardiac events (Fig. 3b; SE-No Sz: 208.6 ± 121.9, SE-Sz: 244.6 ± 85.76, *P* = 0.24). No correlation was found between seizure frequency and total number of arrhythmias on D14 (Fig. 3c, r = −0.062). Lastly, no correlation was found between the number of cortical and hippocampal interictal spikes compared to the number of arrhythmic events (Fig. 3e, r = 0.38; 3 F, r = 0.448).

### Progressive worsening of heart rate instability with each subsequent seizure early following SE

Changes in electrical cardiac function were evaluated to determine if there were any alterations during seizure events and if these observations changed with seizure burden. Example traces for the first (Supplemental Fig. 5a), fifth (Supplemental Fig. 5b), and tenth (Supplemental Fig. 5c) seizures from one SE animal are shown, illustrating progressive HR instability with wider fluctuations in HR over time. Average HR, HR range, HR nadir, and HR coefficient of variance were analyzed during each stage of the first ten seizures for each animal. Seizure stages were defined as: Pre-Ictal (1 min before the beginning of the electrographic seizure), Ictal (the seizure event), Post-Ictal Depression (PID; the time of dampened/flat EEG signal following the seizure), and Post-Ictal (the 1 min following either the end of the seizure or the PID) for each seizure event. HR range was defined as: HR range = max HR - min HR. HR nadir was calculated at the lowest HR. HR coefficient of variance, a measure of relative variability, was determined by dividing the SD of mean by the mean HR.

**Figure 4:**
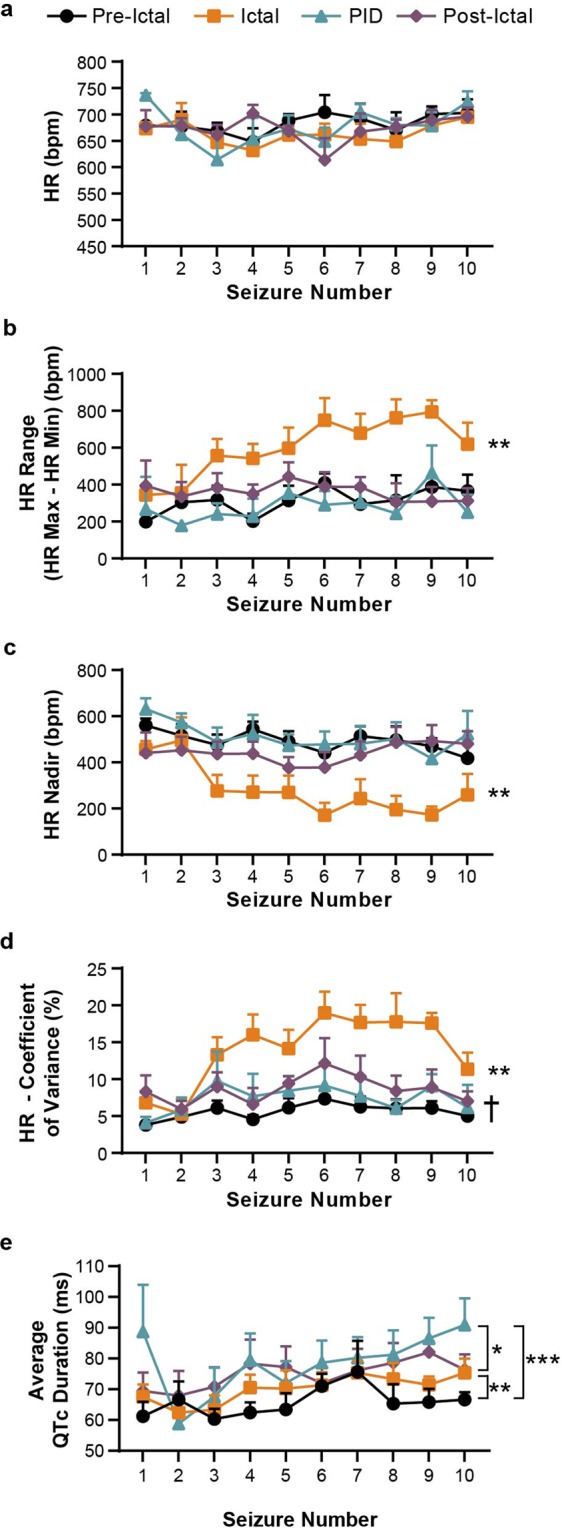
Increased variability of the heart rate occurs with progressive seizures. (**a**) Average HR was not significantly different across the first ten spontaneous seizures or during different seizure stages (*P* = 0.073; mixed-effects analysis). (**b**) The range of HRs during the Ictal period was statistically wider (increased) when compared to the other seizure stages (*P* < 0.0001). (**c**) The HR nadir (lowest value), during the Ictal stage was decreased compared to the other seizure stages (*P* < 0.0001). (**d**) The HR coefficient of variance (relative variability) during the Ictal stage increased across multiple seizures and was increased compared to the other stages (*P* < 0.0001). Additionally, Pre-Ictal HR variance was higher than Post-Ictal (^†^*P* = 0.0009). (**e**) QTc intervals were calculated and compared across seizure stages. The PID stage showed an increased QTc interval duration compared to Pre-Ictal (*P* = 0.0008) and Ictal (*P* = 0.041, mixed-effects analysis) stages. Additionally, Post-Ictal QTc intervals were increased compared to the Pre-Ictal stage (*P* = 0.0024). Comparisons were made using a mixed-effects analysis. All data points represent mean ± SEM (n = 6; *P < 0.05, **P < 0.01, ***P < 0.001).

Quantitative analysis of the first ten spontaneous GTC seizures per animal revealed that the average HR did not change with each progressive seizure (Fig. 4a, *P* = 0.80). Also, for a given seizure, the average HR during each stage of that seizure event was not statistically different from the other seizure stages (Fig. 4a, *P* = 0.35). There was a statistically significant increase in HR range with each progressive seizure during the ictal phase consistent with a wider array of HR fluctuations with each subsequent seizure during this early period of epileptogenesis (Fig. 4b; Ictal vs Pre-Ictal: *P* = 0.0001, Ictal vs PID: *P* = 0.0001, Ictal vs Post-Ictal: *P* = 0.0001). In addition, the HR nadir during the ictal phase gradually decreased with further seizures, suggesting that the wider range of HR was due to a lower HR nadir (Fig. 4c; Ictal vs Pre-Ictal: *P* = 0.0001, Ictal vs PID: *P* = 0.0001, Ictal vs Post-Ictal: *P* = 0.0001). Our data showed an overall higher HR coefficient of variance, an indication of an erratic heart rhythm, during the ictal phase that was exacerbated by further seizures (Fig. 4d; Ictal vs Pre-Ictal: *P* = 0.0001, Ictal vs PID: *P* = 0.0001, Ictal vs Post-Ictal: *P* = 0.0001). Additionally, the post-ictal HR coefficient of variance was higher than pre-ictal variance (Fig. 4d, Pre-ictal vs Post-Ictal: *P* = 0.012). We also evaluated PR, QRS, and QTc intervals. QTc intervals during the PID stage were elevated with each successive seizure when compared to the Pre-Ictal (Fig. 4e, *P* = 0.0008) and Ictal (Fig. 4e, *P* = 0.041) stages. Similarly, Post-Ictal QTc intervals were longer when compared to the Pre-Ictal stage (Fig. 4e, *P* = 0.0024) with successive seizures. In contrast, no differences in PR (Supplemental Fig. 6a, *P* = 0.80) and QRS (Supplemental Fig. 6b, *P* > 0.999) interval duration were observed with subsequent seizures or during each stage.

**Figure 5:**
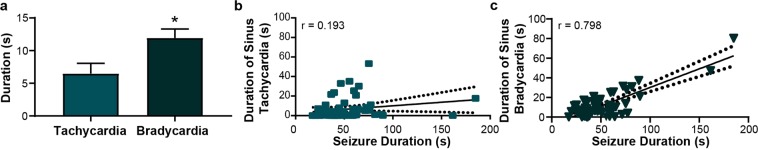
Animals spend more time bradycardic than tachycardic during post-SE seizure events. (**a**) The average duration of bradycardia during the Ictal stage was higher than the duration of sinus tachycardia (n = 60 seizures from 6 animals with 10 seizures each; *P* = 0.011, paired Student’s t-test). Data points represent mean ± SEM (*P < 0.05). (**b**) The duration of sinus tachycardia during the Ictal stage did not correlate with seizure duration (r = 0.193, *P* = 0.14), however, (**c**) the duration of bradycardia during the Ictal stage correlated with an increased seizure duration (r = 0.798, *P* < 0.0001). Each symbol represents an individual ictal event in (**b**,**c**). Regression line and 95% confidence interval are shown.

The time spent at the extremes of the HR range, sinus tachycardia (elevated HR) or bradycardia (lower HR) was calculated, to determine which predominated during a seizure. More time was spent in sinus bradycardia than sinus tachycardia during the ictal phase (Fig. 5a, *P* = 0.0002). As the seizure duration increased, the time that the SE animals spent in tachycardia did not change (Fig. 5b, r = 0.193), however, the duration of bradycardia did increase (Fig. 5c, r = 0.798).

The observed alterations in heart rhythm instability may reflect dysfunction in the autonomic nervous system (ANS). Thus, as one index of ANS dysfunction, HRV measures were taken at baseline, D3, D7, and D14. SE animals demonstrated no significant differences in the high frequency HR component (HF; Supplemental Fig. 7a; Baseline, Veh: 37.66 ± 8.54 nu, SE: 38.15 ± 5.89 nu; D3, Veh: 40.47 ± 7.57 nu, SE: 35.59 ± 4.77 nu; D7, Veh: 43.31 ± 7.70 nu, SE: 50.02 ± 6.09 nu; D14, Veh: 37.83 ± 7.71 nu, SE: 39.75 ± 5.22 nu; *P* = 0.47), the low frequency HR component (LF; Supplemental Fig. 7b; Baseline, Veh: 64.86 ± 8.58 nu, SE: 63.30 ± 6.14 nu; D3, Veh: 60.70 ± 7.85 nu, SE: 65.58 ± 4.94 nu; D7, Veh: 57.72 ± 7.86 nu, SE: 51.18 ± 6.17 nu; D14, Veh: 62.64 ± 7.75 nu, SE: 61.14 ± 5.34 nu; *P* = 0.51), or the LF/HF ratio (Supplemental Fig. 7c; Baseline, Veh: 2.12 ± 0.47, SE: 2.13 ± 0.35; D3, Veh: 1.77 ± 0.38, SE: 2.18 ± 0.30; D7, Veh: 1.60 ± 0.39, SE: 1.32 ± 0.27; D14, Veh: 1.99 ± 0.42, SE: 1.87 ± 0.29; *P* = 0.55). There were no differences in the time domain measures of the root mean square of the successive differences (RMSSD) of the RR interval (Supplemental Fig. 7d; Baseline, Veh: 4.27 ± 0.52, SE: 4.04 ± 0.99; D3, Veh: 5.35 ± 0.65, SE: 4.36 ± 0.83; D7, Veh: 8.23 ± 2.60, SE: 4.62 ± 0.63; D14, Veh: 6.05 ± 0.80, SE: 5.52 ± 0.93; *P* = 0.14) or the percentage of normal consecutive RR intervals differing by greater than 6 ms (pNN6; Supplemental Fig. 7e; Baseline, Veh: 6.61 ± 1.81, SE: 7.38 ± 1.55; D3, Veh: 9.23 ± 1.37, SE: 4.58 ± 1.15; D7, Veh: 12.25 ± 2.12, SE: 5.95 ± 2.25; D14, Veh: 12.25 ± 2.01, SE: 8.65 ± 2.00; *P* = 0.13). None of the HRV measures were different between SE-Sz and SE-No Sz animals. Additionally, we determined if there was a difference before the presence of spontaneous seizures (D3) and after the last recorded spontaneous seizure on D14 in SE-Sz animals and if there was a difference when the lights were on (Light) versus when the lights were off (Dark). There was no difference between D3 and D14 in low frequency, high frequency, and the ratio of low frequency to high frequency power in the light cycle (Light, Supplemental Fig. 8a, *P* = 0.273, 8b, *P* = 0.254, 8c, *P* = 0.073), dark cycle (Dark, Supplemental Fig. 8d, *P* = 0.98, 8e, *P* = 0.978, 8 f, *P* = 0.973), the RMSSD during the light cycle (Supplemental Fig. 8g, *P* = 0.644) or during the dark (Supplemental Fig. 8h, *P* = 0.473), or pNN6 during the light or during the dark (Supplemental Fig. 8i, *P* = 0.976, 8j, *P* = 0.98). We determined if there was a difference in HRV measurements of interictal periods between seizures for SE-Sz animals and the corresponding time points for Veh and SE-no Sz animals. We found no difference in low frequency power (Supplemental Fig. 9a, *P* = 0.424; Veh: *P* = 0.672, SE-no Sz: *P* = 326, SE-Sz: *P* = 0.477), high frequency power (Supplemental Fig. 9b, *P* = 0.314; Veh: *P* = 0.681, SE-no Sz: *P* = 0.254, SE-Sz: *P* = 0.317), the ratio of low frequency to high frequency power (Supplemental Fig. 9c. *P* = 0.168; Veh: *P* = 0.465, SE-no Sz: *P* = 0.23, SE-Sz: *P* = 0.33), RMSSD (Supplemental Fig. 9d, *P* = 0.069; Veh: *P* = 0.456, SE-no Sz: *P* = 0.067, SE-Sz: *P* = 0.53), and pNN6 (Supplemental Fig. 9d, *P* = 0.221; Veh: *P* = 0.613, SE-no Sz: *P* = 0.213, SE-Sz: *P* = 0.257) among Veh, SE-no Sz, and SE-Sz animals and within the respective groups.

### Early mortality post-SE in the intrahippocampal kainate model

**Figure 6:**
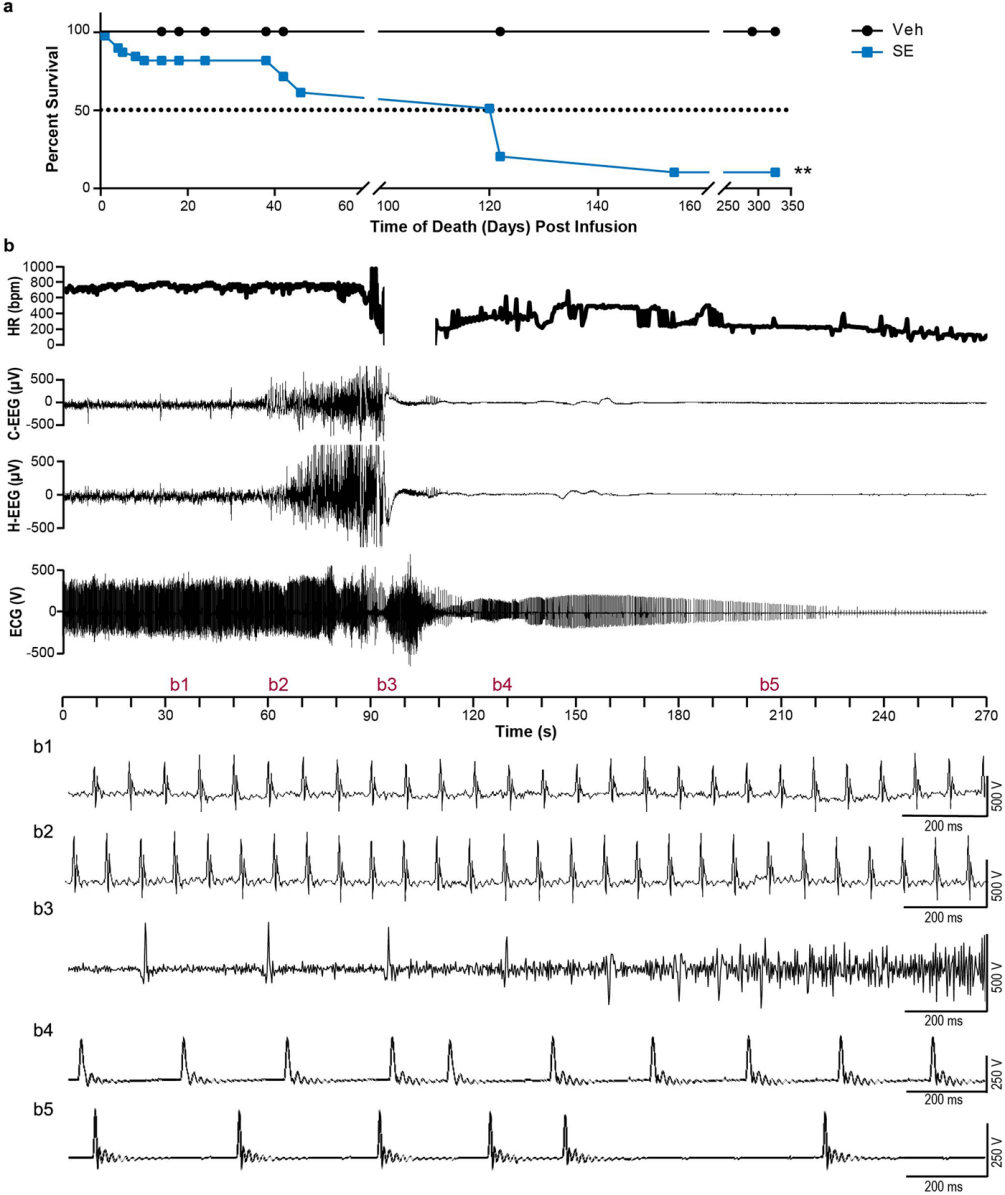
SE animals experienced early mortality and a death event demonstrates bradycardia. (**a**) SE animals (n = 38) had a significantly decreased lifespan compared to that of Veh animals (n = 23; *P* = 0.001; Mantel-Cox Test). (**b**) A death event was captured at D10 post-SE induction as shown here with HR, cortical (C-EEG), hippocampal (H-EEG), and ECG traces. To show changes in the ECG signal throughout the event in more detail, areas of interest have been designated and expanded (b1–b5).

We found that SE animals experienced early mortality compared to Veh animals during the overall 350 d post-induction survival monitoring period (Fig. 6a, *P* = 0.001). At D120, 49% of SE animals had died, and 90% were dead by D154. Most deaths in the SE group occurred after the 14-day monitoring period, however, a single death event associated with a seizure was captured on D10 at 5:34 am in an SE-Sz animal (Fig. 6b). This event was captured on vEEG and provided insight into what contributed to early mortality in this animal. Behaviorally, the seizure started with forelimb tonic-clonic movement and progressed to rearing and falling with full body tonic-clonic activity. The ECG recording of this animal showed a stable sinus rhythm and HR one minute before (Fig. 6b1; 763.63 bpm) and during (Fig. 6b2; 736.36 bpm) the first 10 s of the clonic seizure. Severe sinus bradycardia (Fig. 6b3 and Supplemental Fig. 10; 259 bpm) was observed at 23 s into the seizure. Subsequently, the generalized clonic activity stopped and the animal exhibited full hind-limb tonic extension and the EEG showed suppressed activity in all regions while the ECG at this point showed ventricular escape beats (Fig. 6b4). As the ECG continued to show ventricular escape beats (Fig. 6b5), no further movement was seen from the animal as the heart slowly stopped beating and the EEG remained suppressed. At this point, the animal was still in the same full hind-limb extension position. No other death event was captured while recording vEEG/ECG continuously for 14 d during the early post-SE period, but in the days that followed recording, many SE mice were found dead in their cages with a similar hind-limb extension positioning as seen in the recorded and described death event.

## Discussion

In this study, we characterized HR, cardiac rhythm, and epileptiform activity in a mouse model of SE, which utilized intrahippocampal administration of kainate, thereby avoiding peripheral convulsant drug exposure, while still allowing for SE induction in freely moving and awake animals. In this model, we found an elevated interictal resting HR in SE animals. A significant increase in the number of arrhythmias 14 days following SE was identified, with the majority of events being sinus arrhythmias (SP and SA). Additionally, we observed progressive HR instability ictally and post-ictally in mice that had multiple spontaneous seizures. We found that the duration of ictal bradycardia was positively associated with the duration of seizures.

Hyperactive sympathetic nervous system tone is a common observation during SE, subsequent seizure events, and may be persistently elevated for weeks to months following a seizure^3,10,16–20^. Our finding of elevated interictal HR in the SE animals is consistent with persistently elevated sympathetic nervous system tone. However, a preponderance of sinus arrhythmias as seen in the mice in our study has been associated with parasympathetic nervous system predominance. An increased frequency of epileptiform activity, including seizures, was not correlated with an increase in arrhythmic events in these SE animals, which suggests that at D14 following SE, epileptiform activity is not the primary driver for arrhythmias. These findings support the concept that SE itself may lead to cardiac and brain remodeling. Successive seizure episodes did adversely affect the HR stability, which may indicate ANS dysfunction associated with the development of spontaneous seizures. Furthermore, the duration of bradycardia was positively associated with the duration of seizures. Thus, there may be a higher likelihood of a fatal bradycardic event/asystole during longer duration seizure events. Previous work from other labs suggests that with seizures, asystole and death may occur as a result of severe ictal bradycardia, similar to what was found during the death event recorded in our studies where the HR became progressively slower^3^. Further studies are needed to determine whether frequent seizure events versus SE itself are sufficient to exacerbate ANS instability or if these seizures simply reflect the severity of an altered ANS.

Although our findings of HR alterations and arrhythmic events suggest altered autonomic system control in the SE animals, when we investigated this further through HRV frequency and time domain analyses, we surprisingly found no differences in these measures between Veh and SE animals at baseline, D3, D7, and D14. We have measured HRV, one index of autonomic system dysregulation, but future studies using additional assessments will be important for further assessing autonomic tone. Additionally, there may be mechanisms contributing to our findings in concert or parallel to autonomic alterations. As an example, the sinus nodal dysfunction observed in our study could be due to intrinsic cardiac dysfunction. In humans and experimental models of epilepsy, alterations in a variety of ion channels important for regulating the cardiac action potential and contractility have been described. Previous studies suggest that in epilepsy there may be a switch from a secondary cardiac dysfunction to a primary cardiac dysfunction^4,9,10,21^. Hyperpolarization-activated cyclic nucleotide gated (HCN) channelopathies have been one of the first examples of secondary changes in cardiac ion channel expression due to chronic epilepsy^9,10^. Other cardiac ion channelopathies include, but are not limited to dysfunction or altered expression of adrenergic receptors, T-type calcium channels, and voltage-gated potassium and sodium channels^4,9,10,22,23^. Future studies are necessary to delineate the relative contribution of autonomic instability from intrinsic cardiac remodeling to the observed cardiac phenotypes following SE that we report in our study.

The intrahippocampal kainate mouse model of SE induction provides an opportunity to observe the direct effects of SE as the animals are wildtype at the time of KA administration. Using this model, we have demonstrated interictal sinus tachycardia, an increased number of arrhythmic events, and increased ictal HRV following SE that can be further exacerbated by successive seizure events following SE. These findings, coupled with increased mortality in SE animals, may represent an early risk factor for sudden death following SE. Future studies are needed to further elucidate how cardiac changes progress later in epileptogenesis following SE.

## Methods

### Study design

All procedures complied with and were approved by the Institutional Animal Care and Use Committee of Baylor College of Medicine and conformed to National Institutes of Health guidelines for the Care and Use of Laboratory Animals. Male C57BL/6 J mice (Stock No: 000664; Jackson Laboratory; Bar Harbor, ME, USA) at 2–4 months of age were used for all experiments. Female mice were not used as the estrous cycle is known to affect seizure frequency^24^. Animals were provided food and water *ad libitum* and kept on a 14/10 h light/dark cycle at 22 °C on corncob bedding.

### Surgery and pain management

Cortical and hippocampal depth electrodes were implanted using methods described previously^25,26^. Mice were anesthetized with 2–2.5% isoflurane at 0.5 L/min and positioned in a stereotaxic frame (Stoelting; Wood Dale, IL, USA). EEG electrodes (Plastics One; Roanoke, VA, USA) were implanted relative to bregma as follows: one subdural cortical electrode (0.1 mm posterior, 1.8 mm lateral), a reference subdural electrode anterior to bregma, a hippocampal (CA1) depth electrode (1.6 mm posterior, 1.8 mm lateral, 1.8 mm ventral), and a ground electrode was sutured in the cervical paraspinous region. ECG electrodes were placed on the chest (Type II configuration) and ran subdermally to the top of the head. A guide cannula (1.6 mm; Plastics One; Roanoke, VA, USA) was implanted in the hippocampus (2 mm posterior, 1.8 mm lateral) contralateral to the hippocampal depth electrode^27^. Electrodes and intrahippocampal cannula were held in place with Metabond (Parkell; Edgewood, NY, USA) and dental cement (Co-Oral-Ite Dental Mfg; Diamond Springs, CA, USA). A pain management regimen was provided by veterinarians as follows: carprofen (one 2 mg tablet per day for three days starting the day prior to surgery), slow release buprenorphine (1 mg/kg sc at 1 h prior to surgery), and meloxicam (2 mg/kg ip at 30 min prior to surgery, then every 24 h after for 5 days). Only animals that survived and were monitored continuously during the 14-day period following induction were included in the final analysis.

### Continuous vEEG/ECG recording and induction

Following a one-week recovery period from surgery, animals were habituated in a recording chamber for two days and monitored using the NicoletOne EEG acquisition system (Natus Medical Inc.; San Carlos, CA, USA). Baseline vEEG/ECG activity was recorded for 24 h (Supplementary Fig. 1a). Afterward, freely moving and awake mice were randomly selected for infusion with either kainate (0.4 µg/0.05 µl; ab120100; Abcam; Cambridge, MA, USA) or saline (Veh; 0.05 µl) via intrahippocampal cannula. The placement of the intrahippocampal cannula, injection volume, and spread of vehicle was verified to be located in the CA1 hippocampus (Supplementary Fig. 1b). Kainate-treated animals were monitored for SE using a modified Racine scale to score motor seizures in combination with EEG activity for a minimum 1 h observation period. To capture cardiac and electrographic changes that develop after SE, vEEG/ECG recordings were maintained continuously for 14 days thereafter^28^.

### ECG analysis

Investigators were blinded to experimental group for ECG data analysis. Analyses were performed manually using LabChart v8 software (AD Instruments; Colorado Springs, CO, USA) for the full 24 h periods at baseline, D3, D7, and D14. The following parameters were calculated: mean HR, duration of sinus tachycardia and bradycardia, RR interval, PR interval, QRS interval, QTc interval, HR range, HR nadir, HR coefficient of variance, and HRV measures where indicated^5,29^. Sinus tachycardia and bradycardia during a 24 h period were defined as the average HR being statistically faster or slower, respectively, when compared to the average 24 h HR of Veh animals. To calculate sinus tachycardia and bradycardia during a seizure event, HR standard deviation (SD) was calculated for the 1 min prior to the seizure event. This SD value was then used to calculate 2x above and below the average HR to determine the threshold for sinus tachycardia (2x SD above average HR) and bradycardia (2x SD below average HR). The QTc interval was calculated using Bazett’s formula: QTc = QT/√RR^14^. HR nadir was calculated at the lowest HR during the defined time period. HR range was calculated as: HR range = max HR - min HR. Coefficient of variance was determined by dividing the SD by the mean HR. Arrhythmias and artifacts were removed and HRV calculated using the HRV analysis module in LabChart v8 software. The frequency domain measurements were set as 0.15–1.5 Hz for LF measures and 1.5–5 Hz for HF measures^30^. In the time domain, we used pNN6, meaning we calculated the percentage of normal RR intervals (NN) that differ by more than 6 ms^30,31^. Additionally, RMSSD was calculated as the square root of the mean square successive differences between successive normal intervals^30^.

Arrhythmic events were manually identified and event frequency determined for the 24 h periods at baseline, D3, D7, and D14, with additional arrhythmic event frequencies determined hourly at D14. We found the following arrhythmias (in order of most frequent to least frequent): SA, SP, PAC, and AVB (see Fig. 2b for examples of different arrhythmic events). We defined an SP arrhythmia as a cessation of a heart beat that lasts at least two times longer than the preceding RR interval. Some SPs were long enough to evoke an escape rhythm. SA arrhythmias were defined as a sudden pause in heart rate that was at least 30 ms longer than the preceding RR interval and did not follow a metronomic rhythm.

### EEG analysis

All recordings were digitized (sampling rate: 2000 Hz), filtered (band-pass: 0.5–55 Hz), and reviewed blind to experimental group for epileptiform activity including interictal spikes and seizures using LabChart v8 software. For automated cortical and hippocampal spike detection, the baseline EEG threshold for each recording (baseline, D3, D7, and D14) was manually determined as the best fit amplitude that included the majority of non-epileptiform EEG activity. Spikes were defined as waveforms of negative polarity ≥2.5x the baseline amplitude and within 60 ms in width from the middle of the peak^32^. Artifacts and spiking during seizure events were manually excluded from the spike frequency.

Spontaneous seizures were identified electrographically as having repetitive spiking or spike-and-slow wave activity lasting ≥10 sec and were accompanied by motor behavior. After video confirmation, seizure events were separated into stages as follows: Pre-Ictal (1 min before the beginning of the electrographic seizure), Ictal (the seizure event), Post-Ictal Depression (PID; the time of dampened/flat EEG signal following the seizure), and Post-Ictal (the 1 min following either the end of the seizure or the PID).

### Statistics

Statistics were performed using GraphPad Prism software (La Jolla, CA, USA). The survival curve was compared using the Mantel-Cox test. Quantitative data were analyzed using one-way ANOVA with Turkey’s post-hoc test, two-way ANOVA with Sidak’s post-hoc test, or Student’s t-test as appropriate for the number of groups or variables compared (see figure legends). The significance level was set at *P* < 0.05 and data are presented as mean ± standard error of the mean (SEM).

## Supplementary information

Supplementary information

## Data Availability

The datasets generated and analyzed during the current study are available from the corresponding author on request.
