# Supplementary material for: Cardiac dysregulation following intrahippocampal kainate-induced status epilepticus
Source: Sci Rep. 2020 Mar 4;10:4043. doi: 10.1038/s41598-020-60324-8 (PMC7055295; doi:10.1038/s41598-020-60324-8)
Supplement: Supplementary file 1 — Supplementary information [file 41598_2020_60324_MOESM1_ESM.docx]

**Cardiac dysregulation following intrahippocampal kainate-induced status epilepticus**

Amber T. Levine^1,2,5,6^, Heather A. Born, Ph.D.^2,5,6^, Andrew P. Landstrom, M.D., Ph.D.^7^, Samuel Larson^2,5,6^, Wai Ling Lee, Ph.D.^2,5^, An T. Dao, Ph.D.^2,5,6^, Xander H. Wehrens M.D., Ph.D.^3^, Yi-Chen Lai, M.D.^2^, Anne E. Anderson* M.D.^1,2,4,5,6^

Department of **^1^**Neuroscience, ^2^ Pediatrics, ^3^Molecular Physiology and Biophysics, and ^4^Neurology, Baylor College of Medicine, Houston, TX, USA.

^5^The Jan and Dan Duncan Neurological Research Institute, Texas Children’s Hospital, Houston, Texas, USA

^6^The Gordon and Mary Cain Pediatric Neurology Research Foundation Laboratories, Texas Children’s Hospital, Houston, Texas, USA.

^7^Department of Pediatrics, Division of Cardiology, Duke University School of Medicine, Durham, NC, USA

*Correspondence to [annea@bcm.edu](mailto:annea@bcm.edu)

**Supplemental Figure Legends**

Supplemental Figure 1. Verification of cannula placement, timeline of experiments, representative traces of EEG/ECG at baseline and during status epilepticus (SE) induction, and various cardiac measurements at 1 h and 24 h post-infusion. (a) Relative location of electrodes on the mouse body as well as a dye confirmation of the intrahippocampal cannula location. (b) Experimental outline for continuous EEG/ECG monitoring throughout early post-SE and long-term survival study. (c, d) Examples of cortical EEG, hippocampal EEG, and ECG traces recorded in freely moving mice during (c) baseline and (d) SE induction with expanded ECG traces at c1 and d1. (e-g) Vehicle (Veh; n = 5) and SE (n = 10) animals showed no difference in average duration of (e) PR (*P* = 0.40), (f) QRS (*P* = 0.82), and (g) QTc (*P* = 0.38; two-way ANOVA) intervals at 1 h and 24 h post-infusion of either saline or kainate. All data points represent mean ± SEM.

Supplemental Figure 2. Veh and SE animals demonstrate no alteration in PR, QRS, and QTc intervals over the early post-SE monitoring period. (a-c) Veh (n = 5) and SE (n = 10) animals showed no difference in the average duration of (d) PR (*P* = 0.60), (e) QRS (*P* = 0.56), and (f) QTc (*P* = 0.69; two-way ANOVA) intervals at baseline, D3, D7, and D14. All data points represent mean ± SEM.

Supplemental Figure 3. Veh and SE animals showed no difference in heart rate variability measures during Dark and Light cycles during the recording period. (a-l) Veh (n = 5) and SE (n = 10) animals demonstrated no difference in baseline (a, *P* = 0.84; b, *P* = 0.184; c, *P* = 0.579), D3 (d, *P* = 0.939; e, *P* = 0.915; f, *P* = 0.816), D7 (g, *P* = 0.983, h, *P* = 0.98; i, *P* = 0.926) and D14 (j, *P* = 0.699; k , *P* = 0.7; I, *P* = 0.512; two-way ANOVA) during both when lights are on and when it is dark in the room.. All data points represent mean ± SEM.

Supplemental Figure 4. Spontaneous generalized tonic-clonic (GTC) seizure frequency for individual animals during the early post-SE period.

Supplemental Figure 5. Progressive increase in heart rate (HR) instability with each seizure. (a-c) Representative HR, cortical (C-)EEG, hippocampal (H-)EEG, and ECG traces were recorded in a freely moving mouse during the (a) first, (b) fifth, and (c) tenth spontaneous seizures post-SE. For each seizure, the different seizure event stages are labeled and separated with red lines. Expanded ECG traces are shown during Pre-Ictal (a1, b1, c1), Ictal (a2, b2, c2), Post-Ictal Depression (PID; a3, b3, c3), and Post-Ictal (a4, b4, c4) stages for each seizure.

Supplemental Figure 6. ECG morphology shows no difference between seizure stages or with each subsequent seizure. (a) PR (*P* = 0.80) and (b) QRS (*P* > 0.999; mixed-effects model) interval durations were analyzed during Pre-Ictal, Ictal, Post-Ictal Depression (PID), and Post-Ictal periods for the first ten spontaneous seizures. Measurements were taken during each progressive seizure for each phase of the seizure (n = 6 animals). All data points represent mean ± SEM.

Supplemental Figure 7. SE and Veh animals do not differ in HRV frequency and time domain measures. (a, b) Veh (n = 5) and SE (n = 10) animals showed no difference in the frequency domain measures of (a) high (*P* = 0.47) and (b) low (*P* = 0.51; two-way ANOVA) frequencies in normalized units (nu). (c) The ratio of low/high frequency showed no difference between Veh and SE animals (*P* = 0.55; two-way ANOVA). (d, e) Time domain measures of the (d) root mean square of the successive differences (RMSSD) of the RR interval and (e) the percentage of normal consecutive RR intervals differing by greater than 6 ms (pNN6) showed no difference over time and between Veh and SE animals (*P* = 0.14 and *P* = 0.13 respectively; two-way ANOVA). All data points represent mean ± SEM.

Supplemental Figure 8. Heart rate variability measures demonstrate no difference in post-SE animals before seizure onset (D3) compared to values taken after the last seizure during the monitoring period (D14). Data are shown as a percent change from baseline measurements. There were no significant differences between D3 and D14 (a) in low frequency power during the light cycle (light; *P* = 0.81), (b) low frequency power during the dark cycle (dark; *P* = 0.719), (c) high frequency during the light (*P* = 0.912), (d) high frequency power during the dark (*P* = 0.45), (e) the ratio of low frequency to high frequency during the light (*P* = 0.556), (f) the ratio of low frequency to high frequency during the dark (*P* = 0.647), (g) the RMSSD during light (*P* = 0.147), (h) the RMSSD during the dark (*P* = 0.949), (i) pNN6 during light (*P* = 0.138), or (j) pNN6 during dark (*P* = 0.928). All data points represent mean ± SEM and were analyzed using a two-way ANOVA.

Supplemental Figure 9. Heart rate variability measures during interictal periods between seizures show no differences between Veh, SE-no Sz, and SE-Sz animals. SE-Sz animal HRV measures were taken at interictal time points (at the half way mark between spontaneous seizures). Veh and SE-no Sz animals were analyzed in parallel at corresponding times. No differences were found in low frequency power (a, *P* = 0.424; Veh: *P* = 0.672, SE-no Sz: *P* = 326, SE-Sz: *P* = 0.477), high frequency power (b, *P* = 0.314; Veh: *P* = 0.681, SE-no Sz: *P* = 0.254, SE-Sz: *P* = 0.317), the ratio of low frequency to high frequency power (c, *P* = 0.168; Veh: *P* = 0.465, SE-no Sz: *P* = 0.23, SE-Sz: *P* = 0.33), RMSSD (d, *P* = 0.069; Veh: *P* = 0.456, SE-no Sz: *P* = 0.067, SE-Sz: *P* = 0.53), or pNN6 (e, *P* = 0.221; Veh: *P* = 0.613, SE-no Sz: *P* = 0.213, SE-Sz: *P* = 0.257) between Veh, SE-no Sz, and SE-Sz animals and within the respective groups. All data points represent mean ± SEM and were analyzed using a mixed effects model.

Supplemental Figure 10. Expanded trace of ECG during recorded death event.

(a-d) Continuous cortical EEG (C-EEG), hippocampal EEG (H-EEG), and ECG activity showed ventricular beats and progressive bradycardia occurring during the death event seen in Figure 6. The time on the x-axis is the same as indicated in Figure 6.


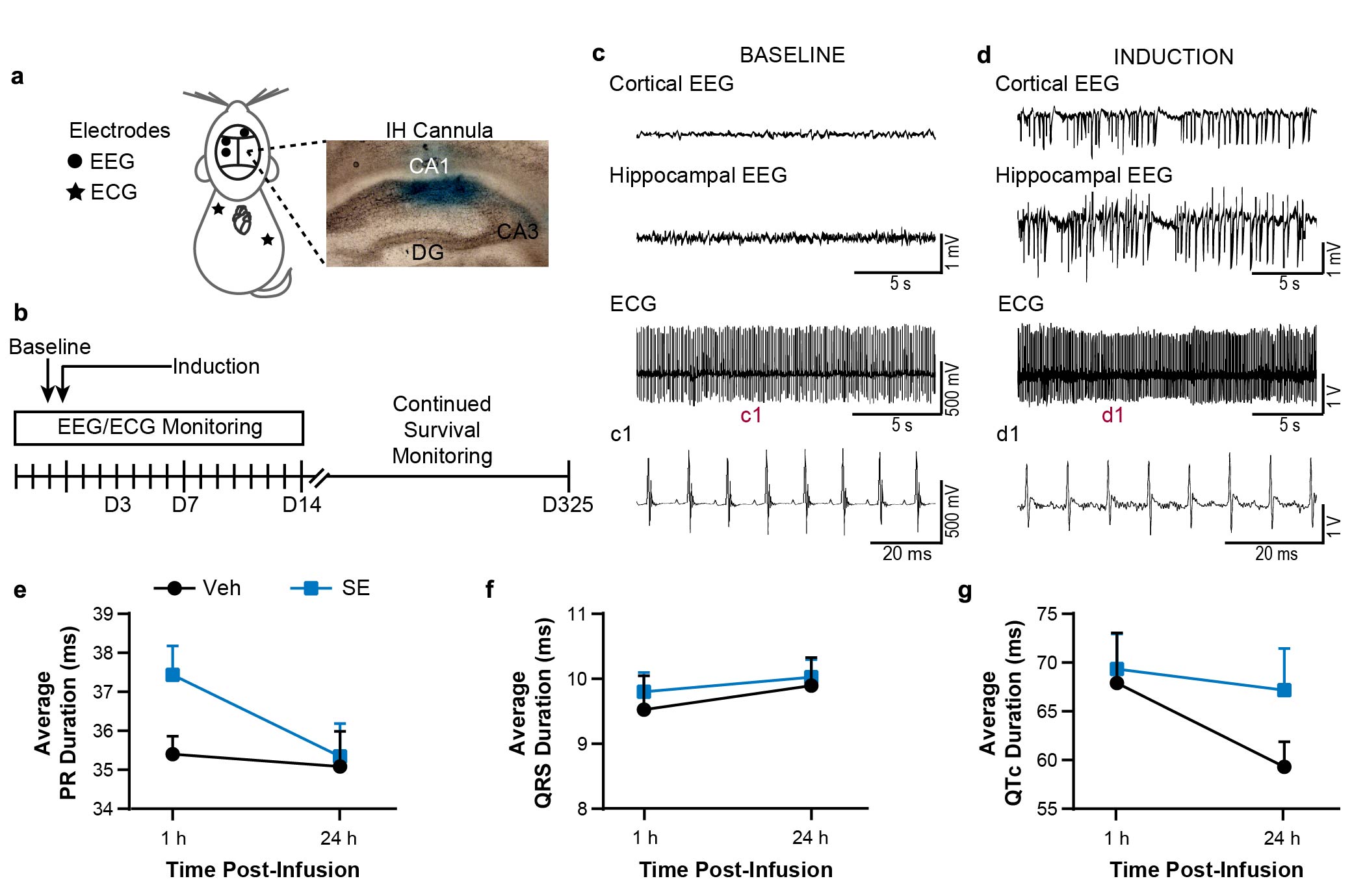
**Figures**

Supplemental Figure 1


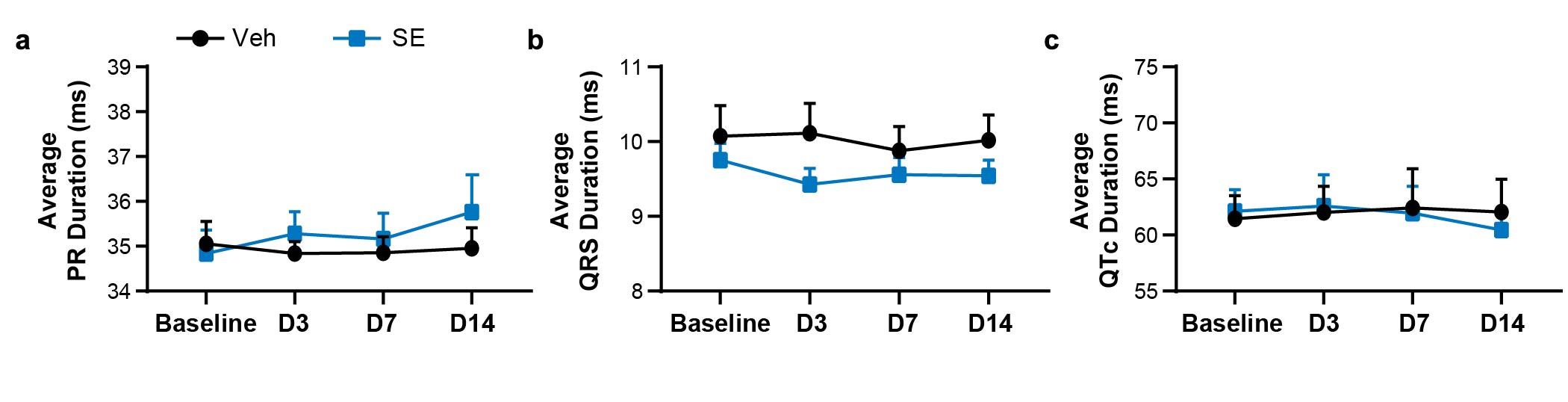


Supplemental Figure 2


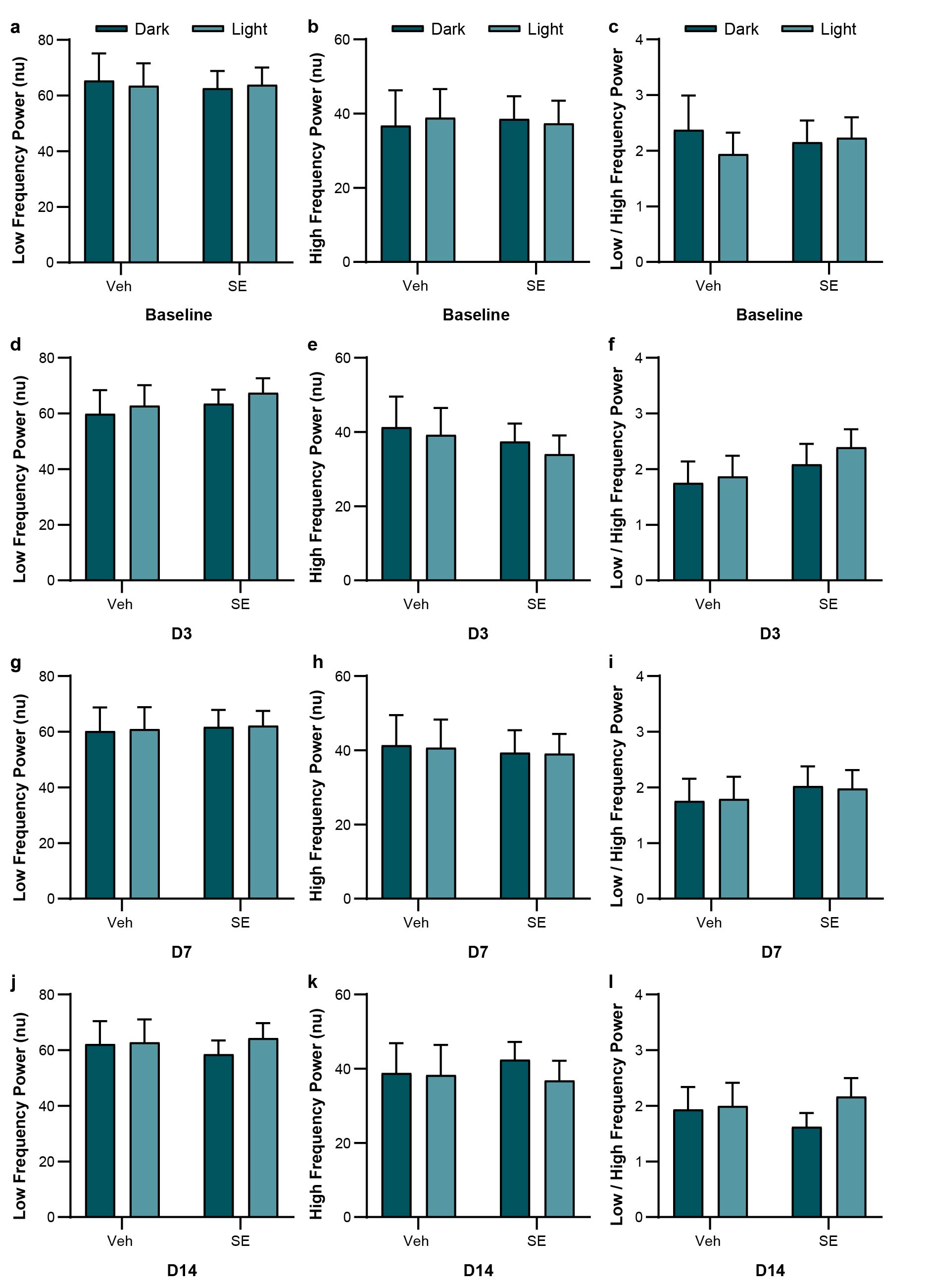
Supplemental Figure 3


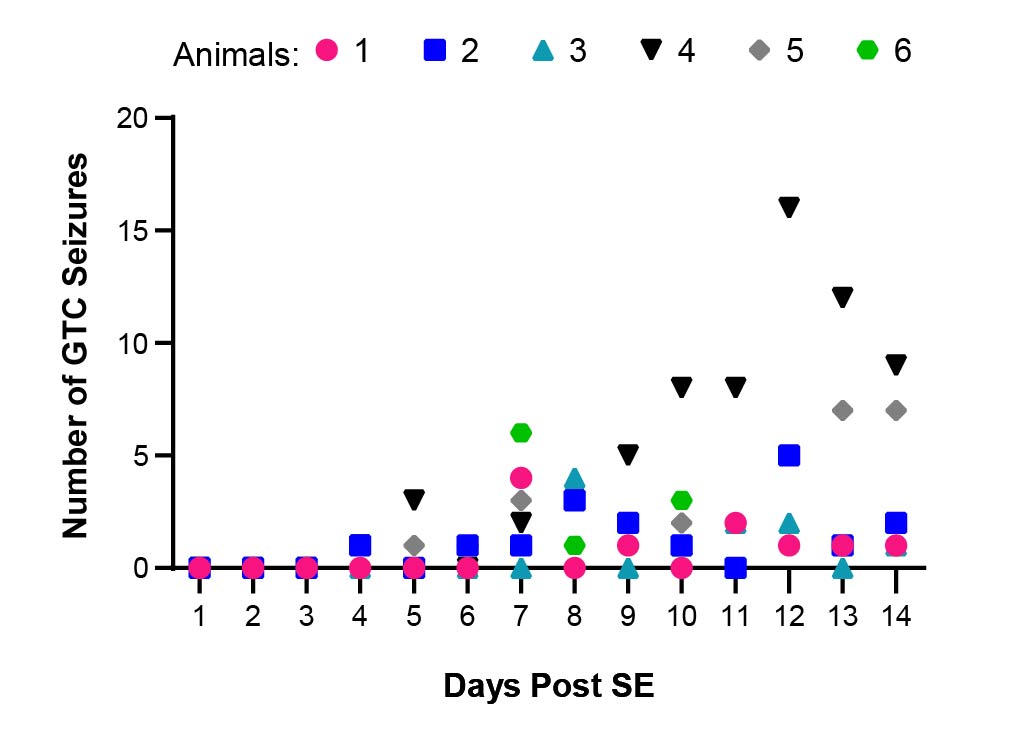


Supplemental Figure 4


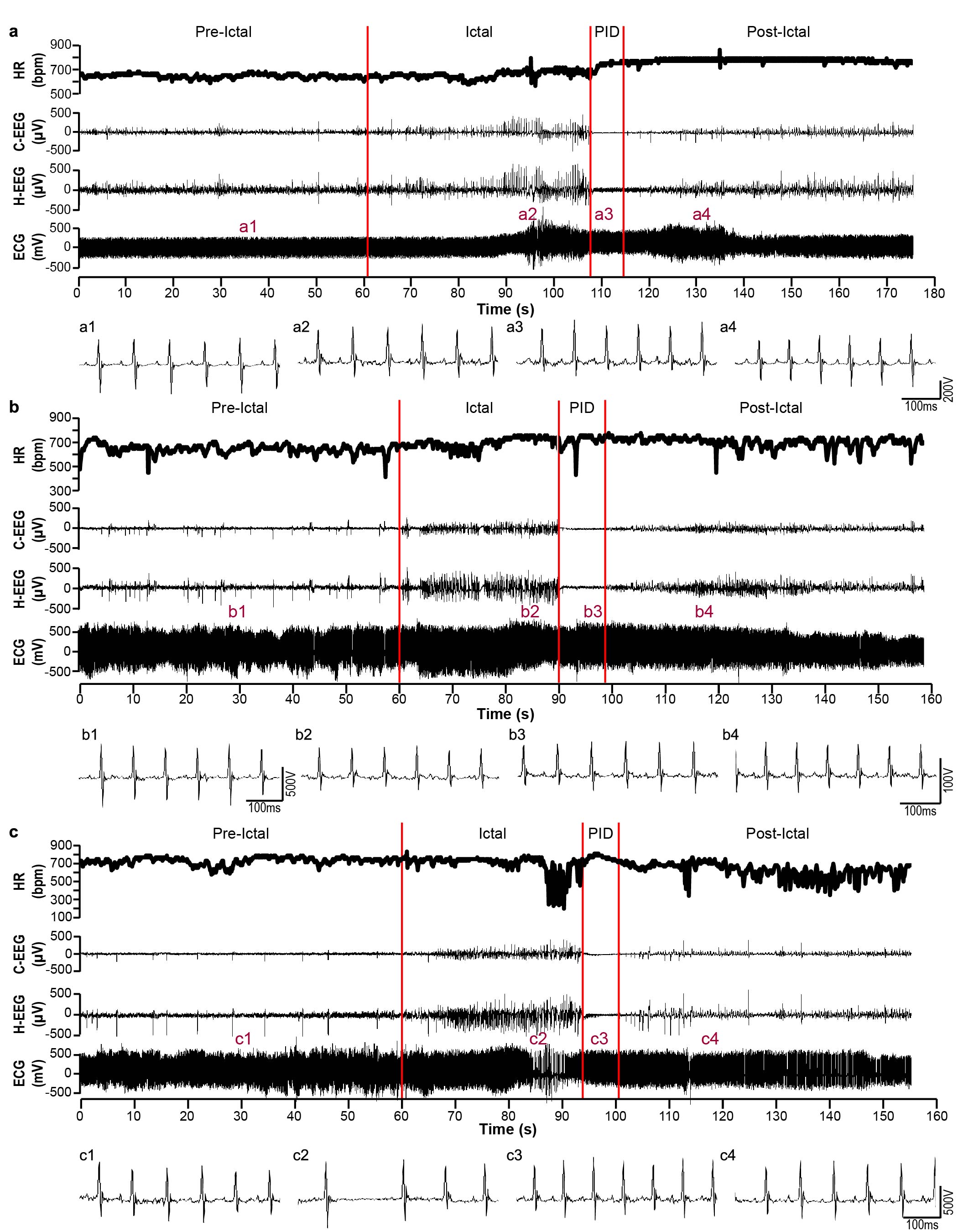


Supplemental Figure 5


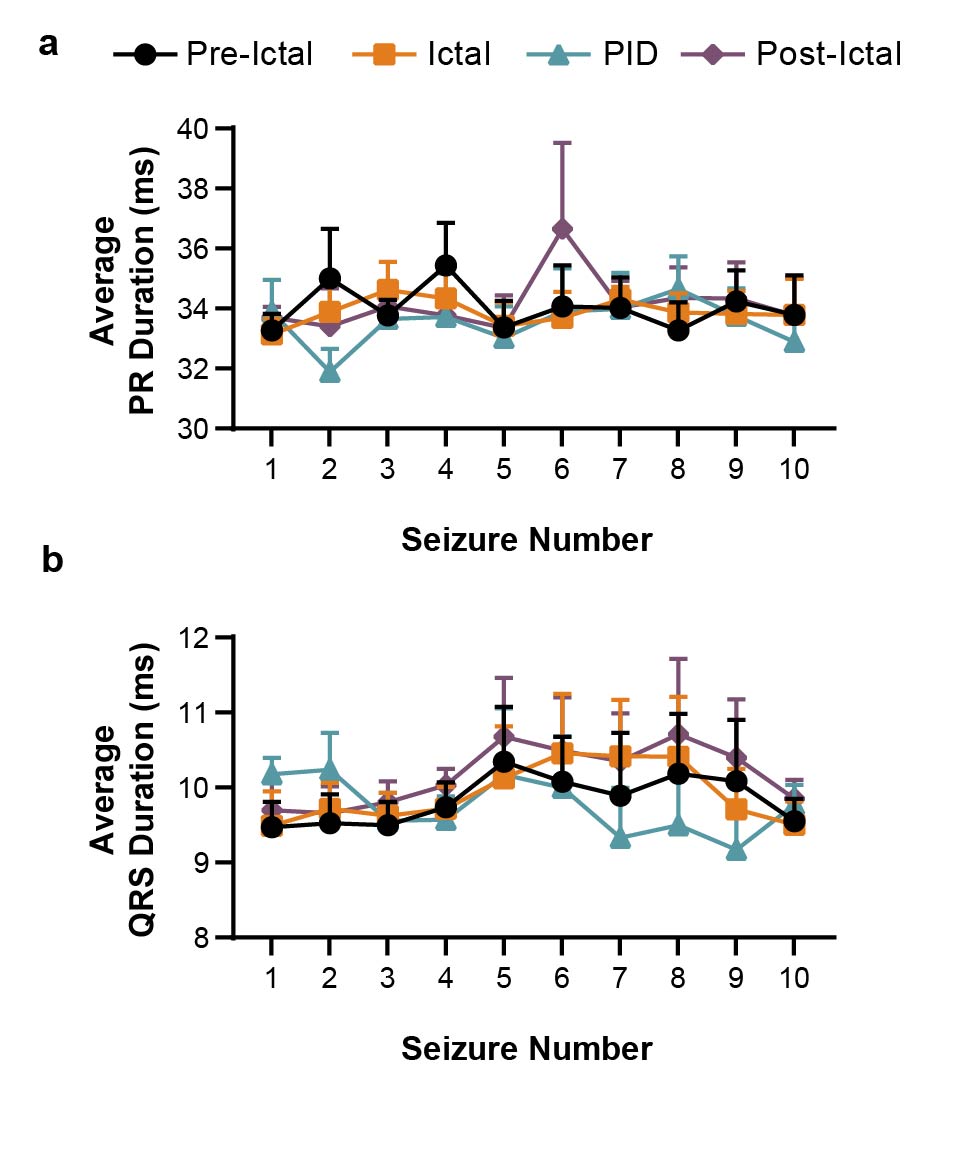
Supplemental Figure 6


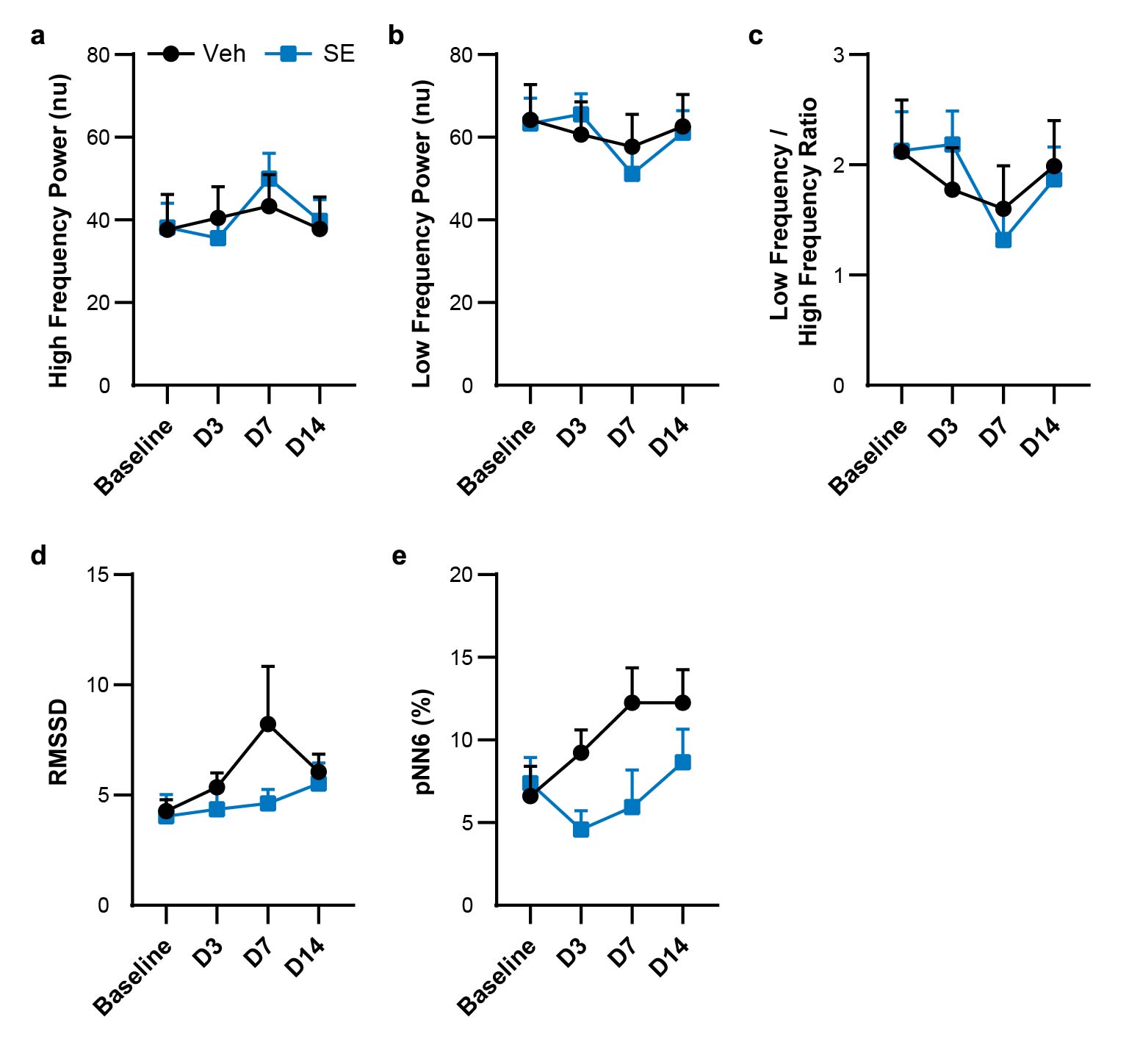


Supplemental Figure 7

Supplemental Figure 8


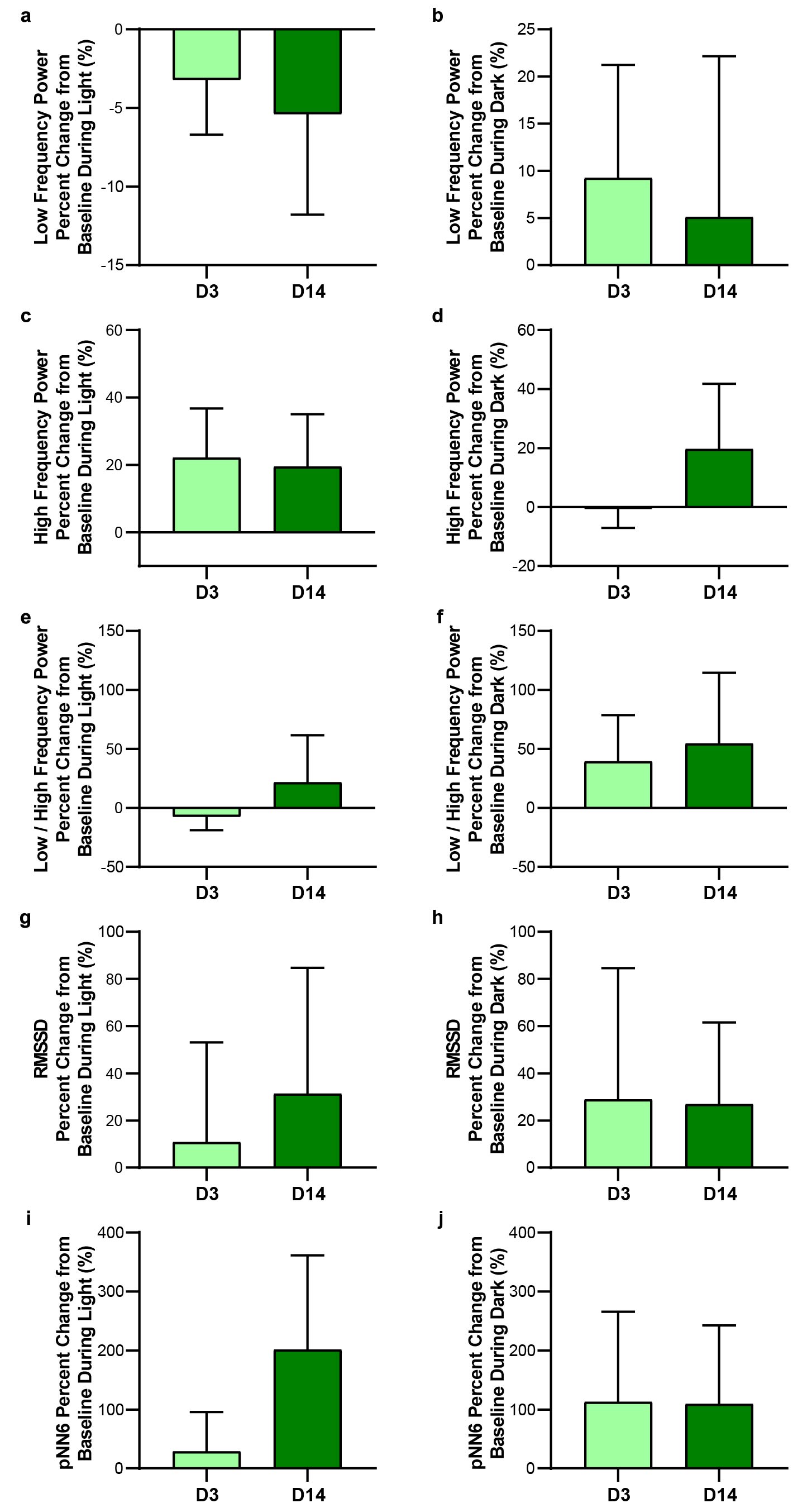

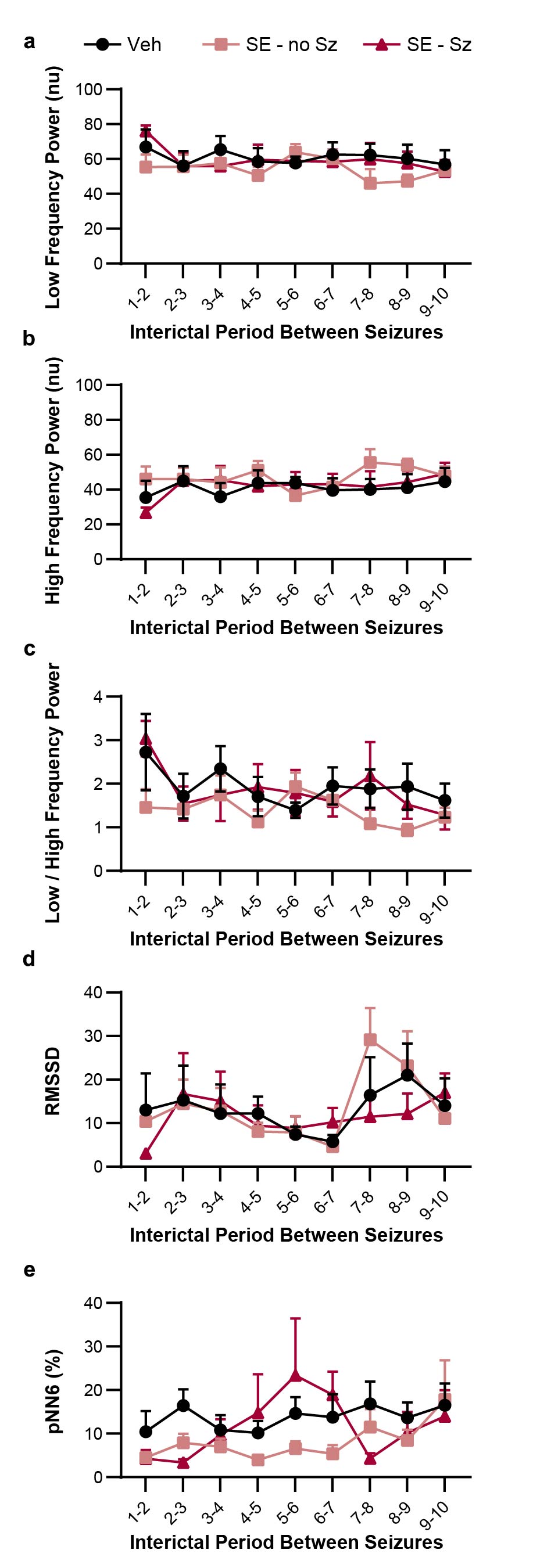


Supplemental Figure 9


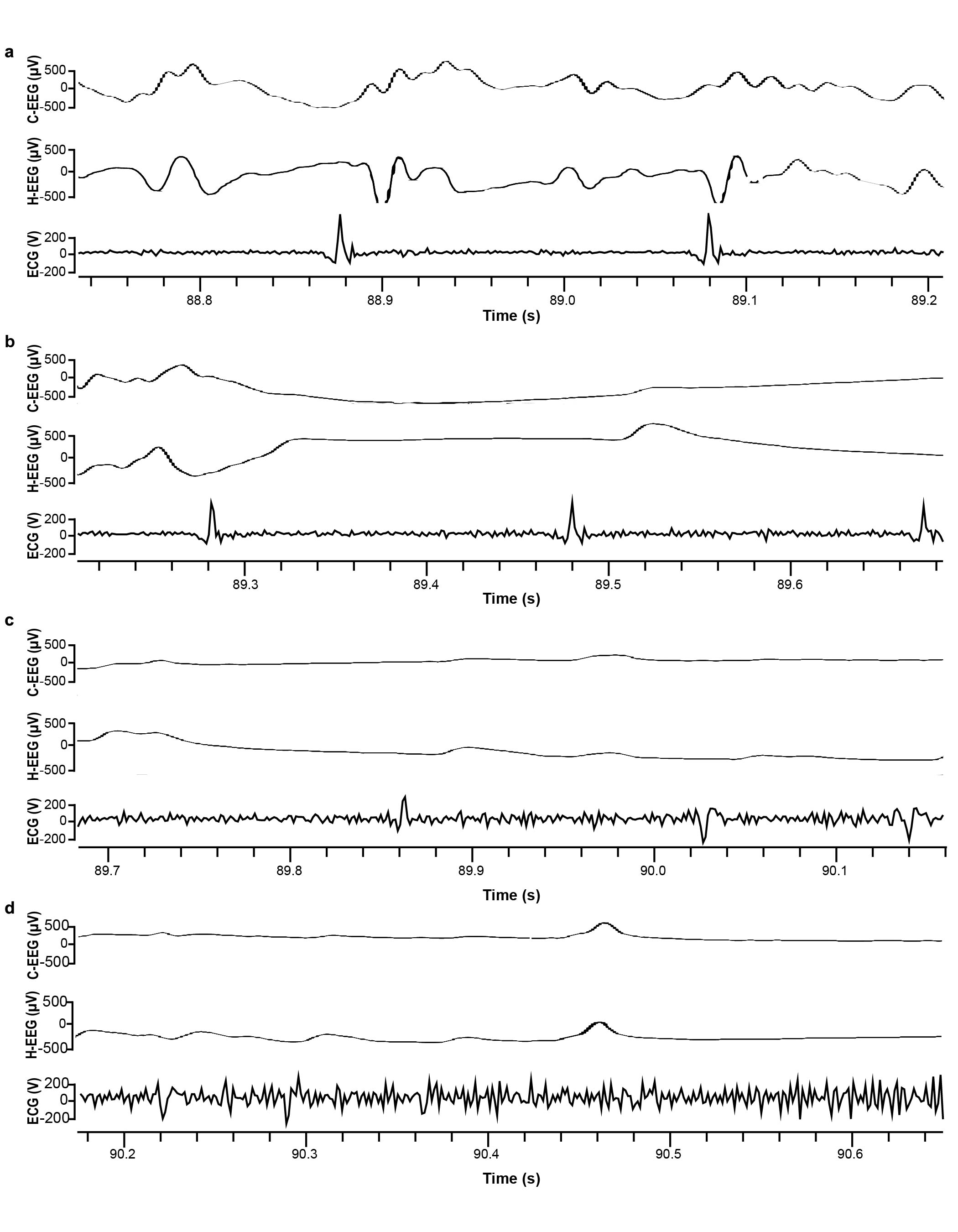
Supplemental Figure 10
